# Stable Isotope Dilution GC/MS for the Quantification of Food Contaminants

**DOI:** 10.6028/jres.093.079

**Published:** 1988-06-01

**Authors:** John Gilbert, James R. Startin

**Affiliations:** Ministry of Agriculture, Fisheries and Food, Food Science Laboratory, Haldin House, Queen Street, Norwich NR2 4SX, UK

## 1. Introduction

As organic contaminants are present in foods at only low levels, and as the food derived extracts for analysis are invariably complex, determinative methods need to be of both a high sensitivity and specificity. Further requirements for an assay are a quantitative ability with a high precision, and the capability of handling large numbers of samples for food surveys. The approach of stable isotope dilution GC/MS fulfills all these requirements; the selected ion monitoring detection method provides high sensitivity and specificity, and the use of stable isotope internal standards enables quantification with relative standard deviations (RSD) of the order of only a few percent. With the use of highly specific mass spectrometric detection, sample clean-up can be minimized, and automated approaches to sample handling and preparation are thus possible, enabling large numbers of samples to be readily analyzed.

Isotope dilution depends on an initial equilibration of the analyte with the stable isotope analogue (usually deuterated or ^13^C-labelled)—this is normally carried out at an early stage by the addition of the internal standard to a solvent slurry of the foodstuff and allowing it to stand for several hours. After equilibration the internal standard will, during extraction, clean-up and sample derivatization, effectively behave in an identical fashion to the analyte, and thus compensate throughout for recovery losses. The main disadvantage of this approach can be the cost of purchasing labelled internal standards, or where they are unavailable the need to carry out possibly lengthy syntheses.

## 2. Contamination of Foods by Migration from Plastic Packaging Materials

Plasticizers such as di-(2-ethylhexyl)adipate (DEHA), and various phthalates are present in both flexible films and in coatings which come into contact with foods during retail packaging or the use of plasticized “cling-film” in the home. Epoxidized soybean oil (ESBO) is used as a plasticizer and secondary heat stabilizer in food packaging and recently so-called polymeric plasticizers have been introduced. These components can migrate from the packaging materials into the foods for which monitoring is therefore necessary. Stable isotope dilution GC/MS methods have proved to be successful for quantification of the levels of migration.

The stable isotope internal standards d_4_-DEHA, d_4_-dibutylphthalate, d_4_-dicyclohexylphthalate and d_4_-diethylphthalate can be readily synthesized from their respective commercially available isotopically-labelled acids, and thereby obtained with high label incorporation and in high chemical purity. Food sample preparation for the determination of these plasticizers involves extraction with acetone/hexane, size-exclusion chromatographic clean-up (automated) in dichloromethane/hexane solvent and a final capillary GC/MS selected ion monitoring quantitative step. For DEHA determinations [1], ions at *m/z* 129 and 133 are monitored for d_0_- and d_4_-DEHA respectively, whilst for the phthalates *m/z* 149 and 153 provide common ions for monitoring a number of unlabelled phthalates and their respective internal standards [2]. The approach has been employed for monitoring migration in a large number of food samples of different types such as cheese, fresh and cooked meat, vegetables, confectionary products and even complete microwave cooked meals [3,4]. In no instance was there any evidence of interference from food components, and the limit of detection of the procedure was 0.1 mg/kg with a RSD of between 1 and 3% [1,2].

So-called polymeric plasticizers are in fact oligomeric mixtures of components, and for example the adipate-based plasticizer “Reoplex R346” has a number average molecular weight some five times greater than the monomeric counterpart. The isotope dilution *GC/MS* procedure [5] for determining polymeric plasticizer levels in foods involves transmethylation of the polymeric plasticizer to dimethyladipate (DMA), utilizing d_4_-DEHA as an internal standard which is in turn transmethylated to d_4_-DMA. Using a similar cleanup to that for the other plasticizers outlined above, the GC/MS determination is based on monitoring of *m/z* 143 and 147 for d_0_- and d_4_-DMA respectively. The method has been demonstrated as applicable to a diverse range of food types, and to have a limit of detection of 0.1 mg/kg and a RSD of about 4%.

ESBO is a multi-functional additive used at levels of between 3 and 7% in a range of plastics. This material is determined in foods by transmethylation of the triglycerides and then derivatization of the epoxide groups in the fatty acid esters to form compounds with characteristics particularly suitable for GC/MS selected ion monitoring. The initial transmethylation is carried out under basic conditions (sodium methoxide/methanol) to ensure protection of the epoxide group, and derivatization involves formation of the 1,3-dioxolane by treatment with cyclopentanone (followed by BF_3_/diethyl ether). The method has been used down to a limit of 0.1 mg/kg without necessitating prior separation of the epoxides from the other naturally occurring fatty acid esters derived from the food lipids. The choice of ions for selected ion monitoring depends on whether the mono-, di- or tri-epoxidized fatty acid esters are to be monitored, but for example for the monoepoxidized species *m/z* 367 and 396 are detected. The isotopically-labelled internal standard is easily synthesized from commercially available ^13^C-triolein, to give a method with an overall RSD of about 5%.

## 3. Pesticide Residues in Foods

The determination of pesticide residues in foods by stable isotope dilution presents some difficulties, firstly because of the large number of compounds that have to be simultaneously monitored, and secondly the high proportion of samples containing not detectable levels of pesticides which makes quantification the exception rather than the rule. For these reasons it is probably preferable to initially screen by another method and then re-analyze positive samples by GC/MS adding only the appropriate labelled internal standards for which quantification is required. For organochlorine pesticides at least 15 deuterated and/or ^13^C-labelled standards are commercially available, but these surprisingly have not been utilized for residue analysis and to date our own experience has been limited to the quantification of hexachlorobenzene (HCB) using the ^13^C_6_-labelled standard [6]. For the analysis of eggs, sample preparation involves initial treatment with phospholipase, extraction with acetone/hexane and then clean-up of the lipid extract on a water deactivated alumina column. Selected ion monitoring is for *m/z* 284 and 286 for HCB and 292 and 294 for [^13^C_6_]HCB. The GC/MS program involves switching between a number of different ions, grouped for different retention time windows to enable monitoring of altogether 10 organochlorine pesticides plus respective isomers. For the future it is intended to increase the number of isotopically labelled standards utilized which will further increase the complexity of the multiple ion monitoring program.

## 4. Veterinary Drug Residues in Animal Tissue

Veterinary drug residues in foods, although obvious candidates for isotope dilution approaches to analysis, do present the same logistical problems as pesticides with the same requirement for multiresidue monitoring. For the determination of the sulphonamide drug, sulphamethazine in kidney samples by isotope dilution GC/MS [7], after addition of d_4_-sulphamethazine to an acetonitrile slurry of the kidneys and allowing for equilibration, the clean-up involves solvent partition, diazomethane treatment to form the methyl derivative and then HPLC fractionation as a further clean-up stage. In the trapped HPLC fraction GC/MS selected ion monitoring for *m/z* 227 and 228 for sulphamethazine and 231 and 232 for the deuterated internal standard is carried out for quantification. The limit of detection of the method is around 0.05 mg/kg and the CV is between 3.7 and 5.7% for sulphamethazine spiking levels in the tissue from 0.2 to 1.2 mg/kg. Deuterated sulphamethazine had to be custom synthesized and other stable isotope-labelled drugs are not widely available commercially which hinders development of this approach, as does the fact that many drugs of interest are not amenable to GC. One possible way to overcome this latter difficulty is to maintain the use of isotope dilution but to use MS/MS which also confers advantages of reduced sample preparation and cleanup [8].
